# P-859. Seeing Beyond the Culture: Smarter UTI Diagnosis in Spinal Cord Injury Patients

**DOI:** 10.1093/ofid/ofaf695.1067

**Published:** 2026-01-11

**Authors:** Maria D Morel Almonte, Deborah Jimenez Jerez, Beatriz Restrepo, Paola Lichtenberger

**Affiliations:** Jackson Memorial Hospital/ University of Miami, Miami, FL; University of Miami/Jackson Memorial Health Sytem, Miami, FL; Miami VA, Miami, Florida; University of Miami Miller School of Medicine and the Miami VA Healthcare System and University of Miami, Miami, FL

## Abstract

**Background:**

Diagnosing urinary tract infections (UTIs) in individuals with spinal cord injury (SCI) presents unique challenges due to atypical clinical presentations, which stem from altered sensation and varied bladder management strategies. This population also faces disproportionately high rates of asymptomatic bacteriuria (ASB) and multidrug-resistant organisms (MDROs), largely due to frequent bladder instrumentation, colonization, and repeated antimicrobial exposure. As a result, UTIs are often misdiagnosed or overtreated, leading to unnecessary antimicrobial use and increased risk of resistance. Improving diagnostic accuracy and antimicrobial stewardship is therefore crucial in the SCI population.

**Methods:**

This quality improvement project was implemented in three phases starting July 2024: (1) baseline assessment of UC and antimicrobial prescribing practices, (2) introduction of a standardized UC order set in the electronic medical record (Figure 1) with provider education, and (3) post-intervention monitoring of diagnostic and prescribing patterns.

**Results:**

Following the July 2024 intervention, adherence to the UC order set among SCI providers reached 93.8%. There was a notable reduction in the number of urine cultures ordered. Although a higher number of UTI cases were identified in newly admitted patients, there was also a significant increase in interventions to tailor antimicrobial therapy based on culture results, indicating improved alignment with diagnostic findings. Additionally, fewer ASB cases were treated with antimicrobials, suggesting improved clinician awareness and adherence to stewardship principles following the educational component.

Please see Tables 1 and 2 to appreciate our results.

**Conclusion:**

The implementation of a standardized urine culture order set, coupled with targeted provider training, led to improved diagnostic practices and more judicious antimicrobial use in the SCI unit at the Miami VAMC. These findings underscore the importance of combining system-level interventions with clinician education to enhance UTI diagnosis and treatment in complex patient populations.

**Disclosures:**

All Authors: No reported disclosures

